# Supplementary material for: A non-canonical lymphoblast in refractory childhood T-cell leukaemia
Source: Nat Commun. 2025 Nov 12;16:9397. doi: 10.1038/s41467-025-65049-8 (PMC12612194; doi:10.1038/s41467-025-65049-8)
Supplement: Supplementary file 1 — Supplementary Information [file 41467_2025_65049_MOESM1_ESM.pdf]

Supplementary Fig. 1

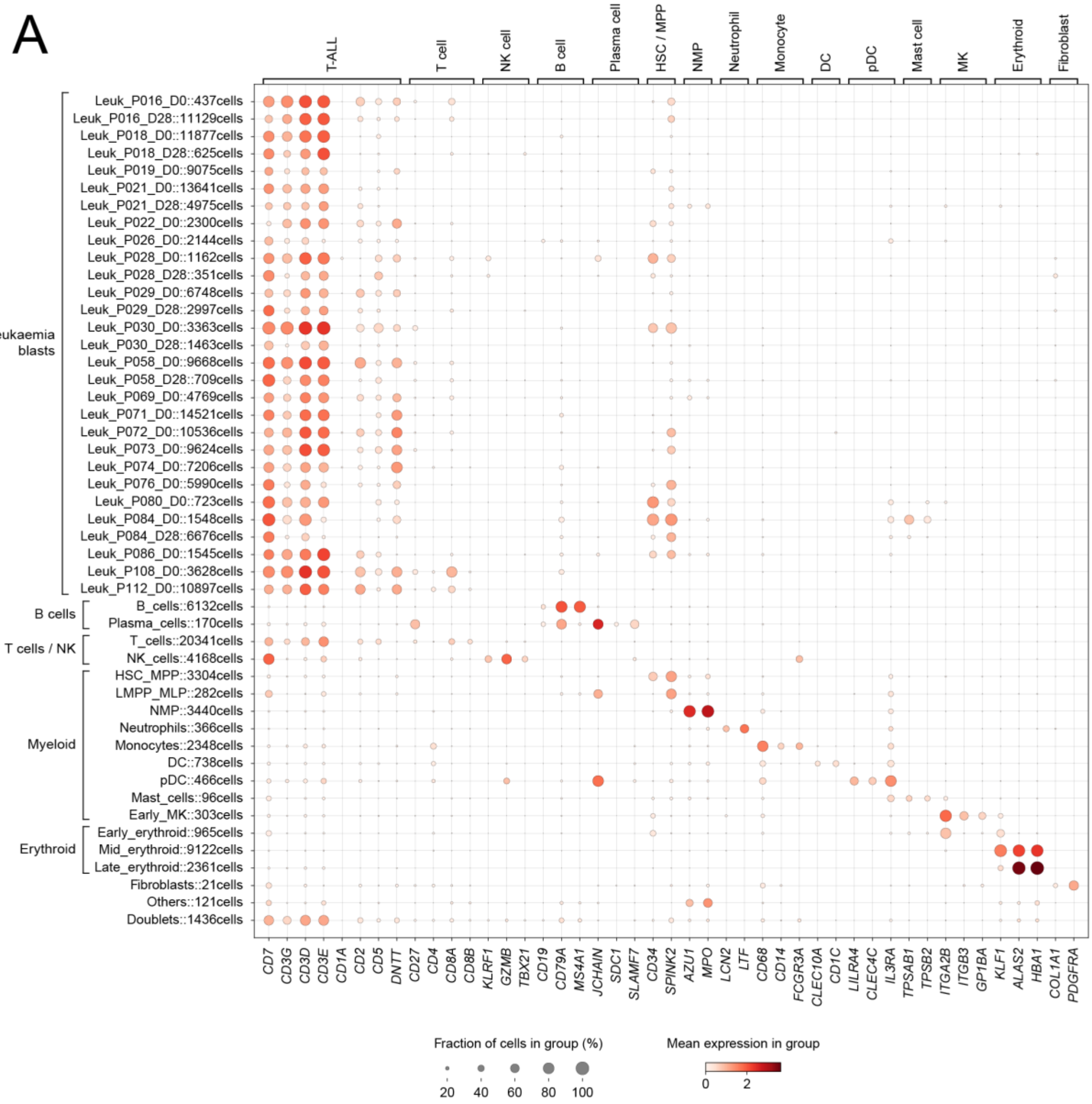

Supplementary Fig. 1: Cell annotation of original scRNA-seq.

(**A**) Dotplot showing the expression of canonical markers to support annotation of cell clusters. Dot size corresponds to the fraction of cells in each group that expresses each gene and colour intensity indicates the mean expression. Leukaemia blasts ("Leuk") are split into separate rows for each sample, defined by patient and timepoint. Normal cells are divided into fine-level annotations, where brackets around normal cell rows indicate cell type category as seen in **Fig. 1C**. Total cell number for each row is denoted in the row label.

NK, natural killer cell; HSC, haematopoietic stem cell; MPP, multipotent progenitor; LMPP, lymphoid-primed multipotent progenitor; MLP, multi-lymphoid progenitor; NMP, neutrophil-monocyte progenitor; DC, dendritic cell; pDC, plasmacytoid dendritic cell; MK, megakaryocyte.

Supplementary Fig. 2

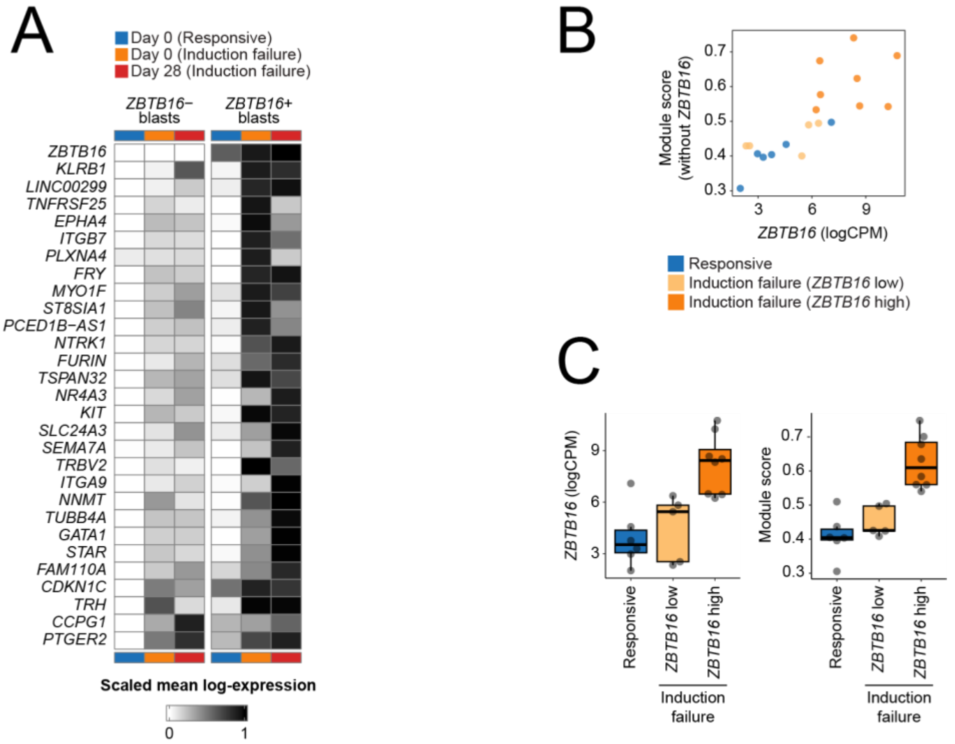

Supplementary Fig. 2: Derivation of gene module of non-canonical blasts.

(**A**) We derived a 29-gene module for *ZBTB16*+ blasts using a pseudo-bulk approach that represents the non-canonical blast state. Here, the heatmap is showing the expression of module genes across *ZBTB16*+ and *ZBTB16*− blasts, taken from day 0 samples of responsive patients (blue), from day 0 samples of induction failure patients (orange), and from day 28 samples of induction failure patients (red). Expression values are log-normalised gene expression averaged across blasts within each group.

(**B**) Using diagnostic bulk transcriptomes from the same patients in the original scRNA-seq cohort, we plot *ZBTB16* module score (without *ZBTB16*) against *ZBTB16* expression, to show an association between the module genes and *ZBTB16*. Scatter plot points are coloured by whether a sample was from a responsive patient (blue) or a patient with induction failure that was identified as *ZBTB16* low (yellow) or *ZBTB16* high (orange) based on Figure 1F.

(**C**) Boxplots showing *ZBTB16* expression and module score in diagnostic bulk transcriptomes as seen in (**B**).

Supplementary Fig. 3

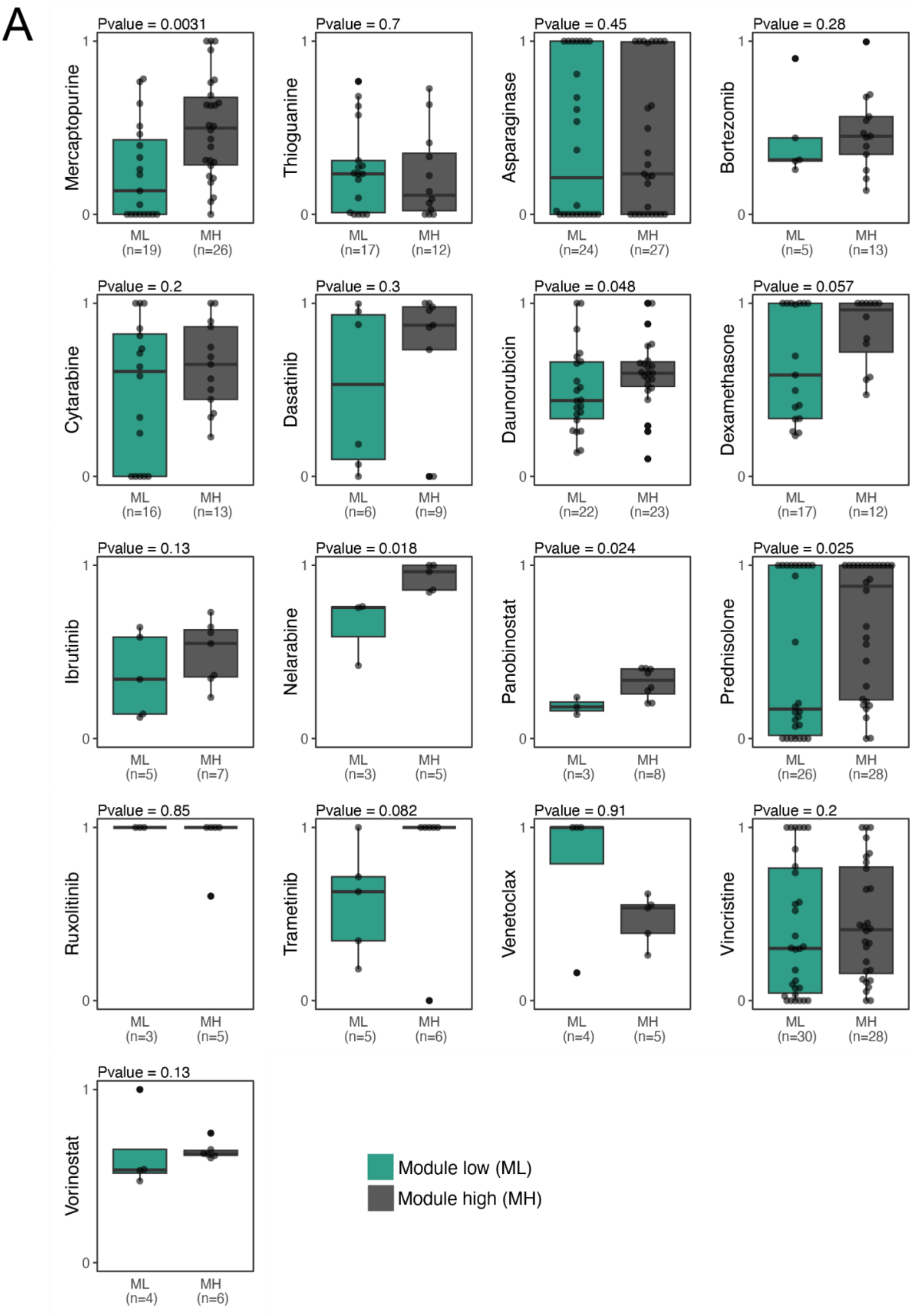

Supplementary Fig. 3: Therapeutic vulnerabilities of non-canonical blasts.

(**A**) Boxplots show 50% lethal concentrations (LC50) of T-ALL primary blast cultures treated with various therapeutic agents in a previously published ex vivo drug sensitivity screen. Samples were stratified into top (module high / "MH") and bottom 30% (module low / "ML") module scores of their bulk transcriptomes. *P* values indicate significance according to one-sided Wilcoxon rank-sum test, testing if MH samples have elevated LC50 values (indicating drug resistance) compared to ML samples.

Supplementary Fig. 4

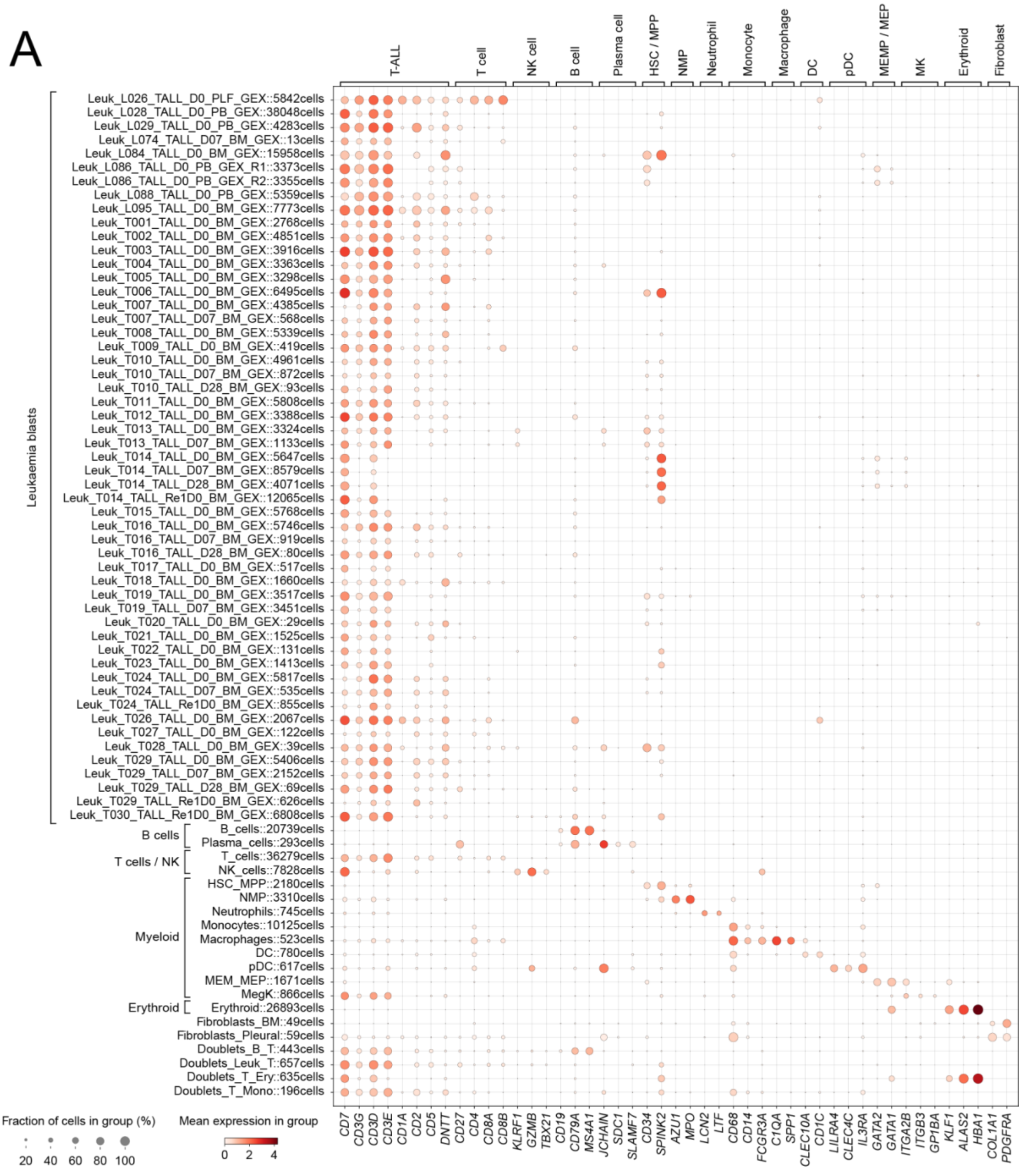

Supplementary Fig. 4: Cell annotation of validation scRNA-seq.

(**A**) Dotplot showing the expression of canonical markers to support annotation of cell clusters. Dot size corresponds to the fraction of cells in each group that expresses each gene and colour intensity indicates the mean expression. Leukaemia blasts ("Leuk") are split into separate rows for each sample. For one patient (L086), two technical replicate samples were obtained from the diagnostic peripheral blood. Normal cells are divided into fine-level annotations, where brackets around normal cell rows indicate cell type category as seen in **Fig. 3A**. Total cell number for each row is denoted in the row label.

NK, natural killer cell; HSC, haematopoietic stem cell; MPP, multipotent progenitor; NMP, neutrophil-monocyte progenitor; DC, dendritic cell; pDC, plasmacytoid dendritic cell.

Supplementary Fig. 5

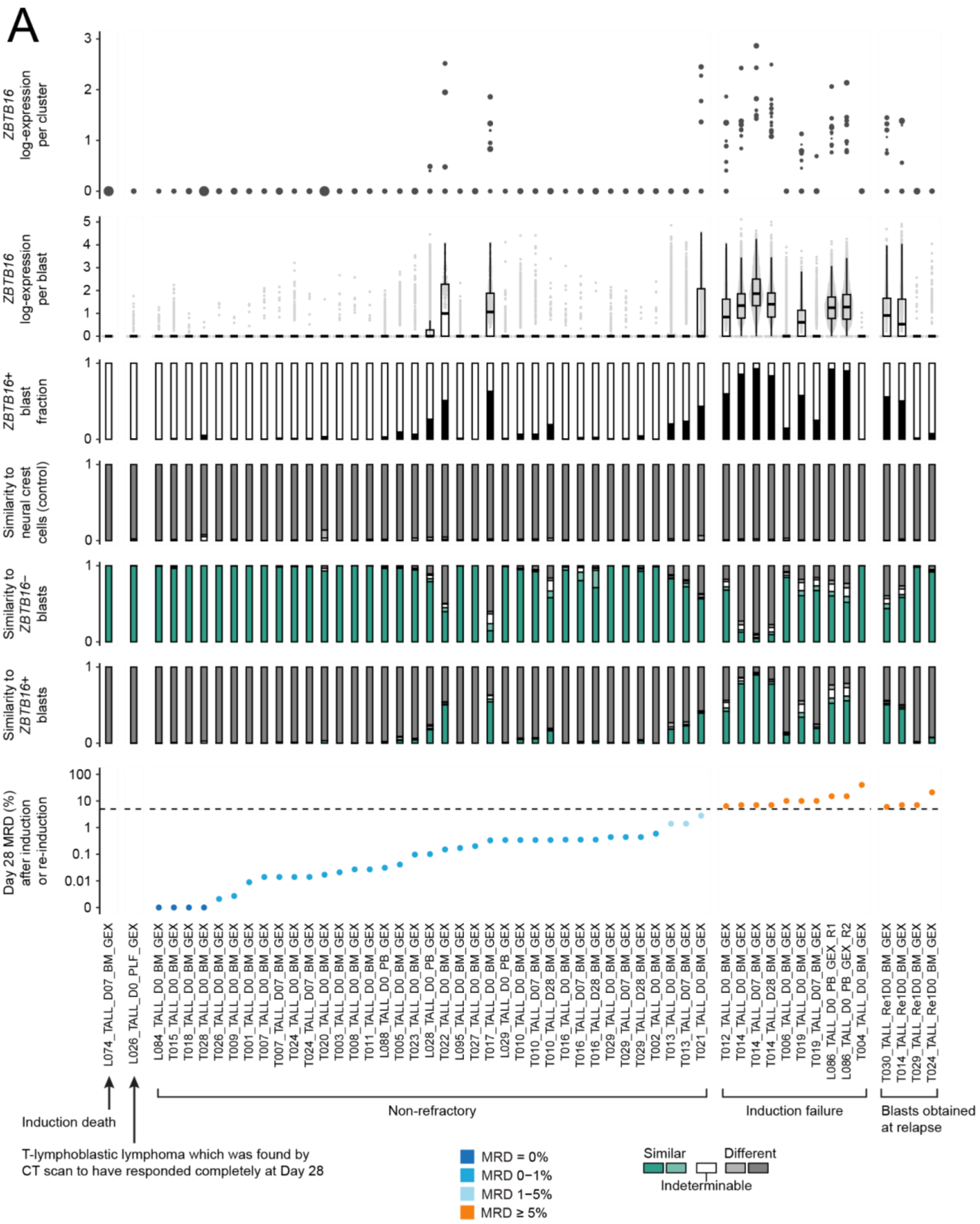

Supplementary Fig. 5: Unbiased cell matching of validation scRNA-seq

(**A**) Unbiased cell matching and *ZBTB16* expression of blasts from validation scRNA-seq cohort. Panel 1 (from bottom): Day 28 MRD (%), coloured by MRD group. Panels 2, 3 and 4: A logistic regression model was trained using *ZBTB16*+ blasts and *ZBTB16*− blasts from the discovery cohort, as well as neural crest cells which serve as control; this model was used to determine the similarity of blasts from the validation cohort. Panel 5: Fraction of blasts expressing *ZBTB16*. Panel 6: Boxplot of *ZBTB16* expression at single cell resolution. Panel 7: Median *ZBTB16* expression of each cluster of blasts within each sample; size of circle indicates cluster size. Two technical replicates were performed on the day 0 peripheral blood sample of L086.

Supplementary Fig. 6

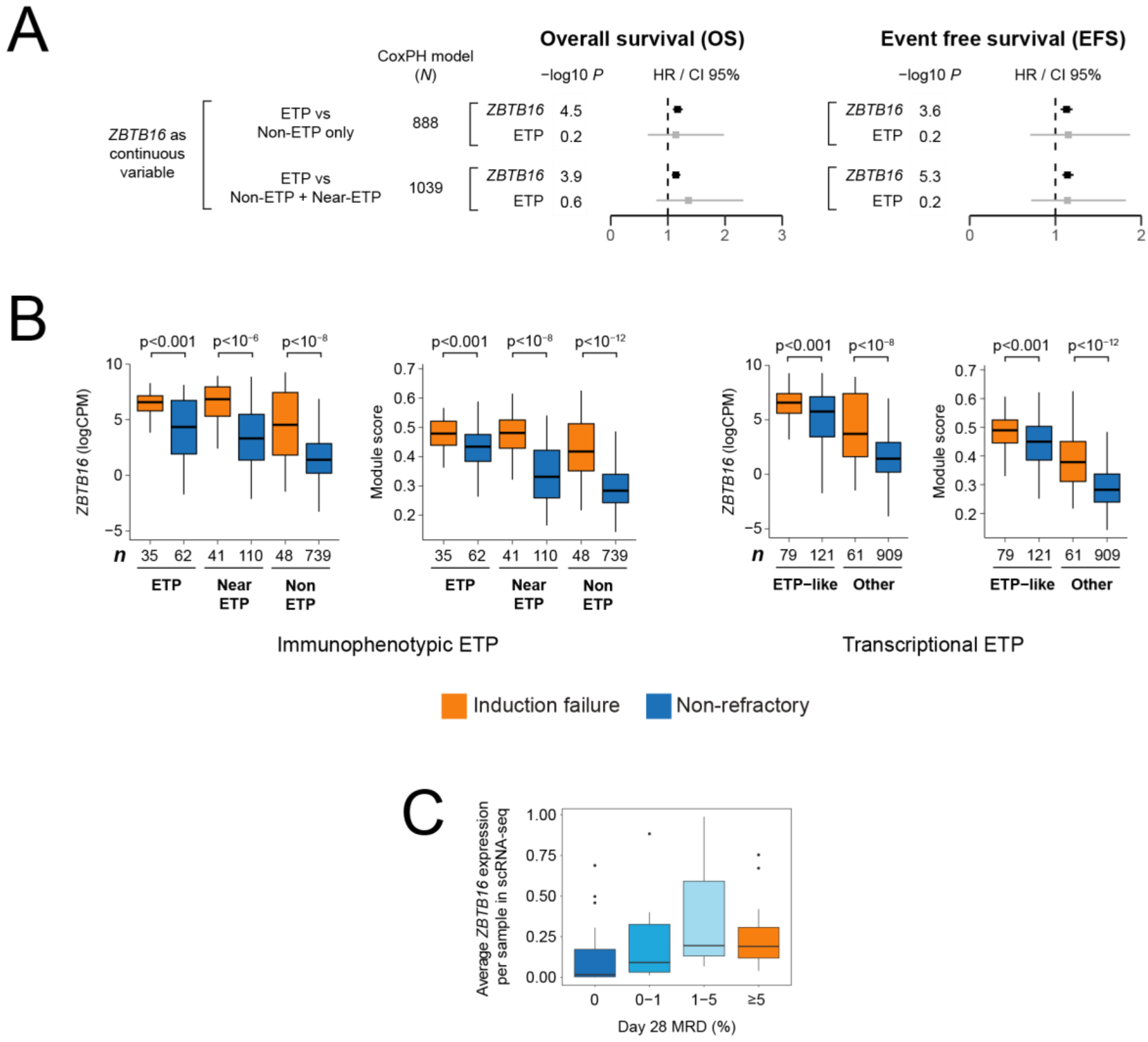

Supplementary Fig. 6: Relationship between the presence of *ZBTB16*+ blasts, ETP status and clinical outcome in the COG ALL0434 cohort.

(**A**) Hazard ratios (HR) and 95% confidence intervals (CI 95%) for various Cox proportional hazard (CoxPH) models of overall survival and event free survival using the published COG AALL0434 study. Both *ZBTB16* and immunophenotype-defined ETP status were tested as variables, where *ZBTB16* considered as a continuous variable and ETP status was considered either excluding or including the "Near-ETP" label. Number of individuals in each CoxPH model is indicated by '*N*'. Statistically significant hazard ratios ($P < 0.05$) in black and non-significant ones in grey.

(**B**) Boxplots showing *ZBTB16* expression and module scores in the COG ALL0434 cohort, grouped by ETP status (either immunophenotypic or transcriptionally defined) and response to induction. We compared signals of non-canonical blasts (*ZBTB16* expression and module score) to both definitions of ETP: ETP reported by flow cytometry (left) and a novel transcriptional subtype "ETP-like" (right). Signals of non-canonical blasts resolved two weaknesses of ETP as a diagnostic marker of high-risk T-ALL. Firstly, high *ZBTB16* expression and module score identified children with "ETP negative" disease at diagnosis who were refractory after induction treatment. At the same time, the absence of signals of non-canonical blasts identified children with responsive T-ALL erroneously deemed high-risk by ETP status.

(**C**) Boxplots showing average *ZBTB16* scaled-expression per patient across MRD groups using the scRNA-seq data from the authors that derived the "BMP-like" signatures.

Supplementary Fig. 7

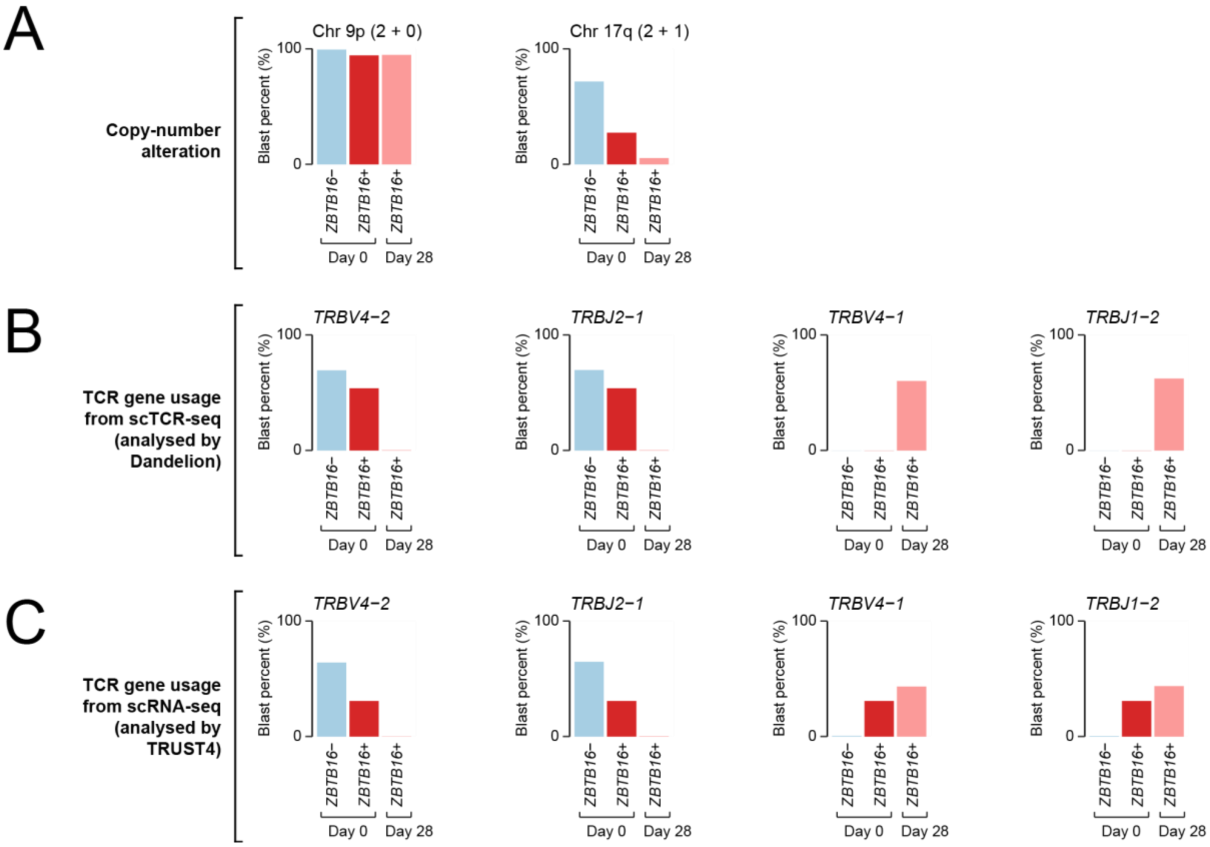

Supplementary Fig. 7: Phylogenetic features in patient P058.

(**A**) Percentage of P058 blasts harbouring the chromosome 9 and chromosome 17 copy number alteration.

(**B**) Percentage of P058 blasts expressing specific TCR genes determined from Dandelion analysis of scTCR-seq data.

(**C**) Percentage of P058 blasts expressing specific TCR genes determined from TRUST4 analysis of scRNA-seq data.

TCR, T-cell receptor.

Supplementary Fig. 8

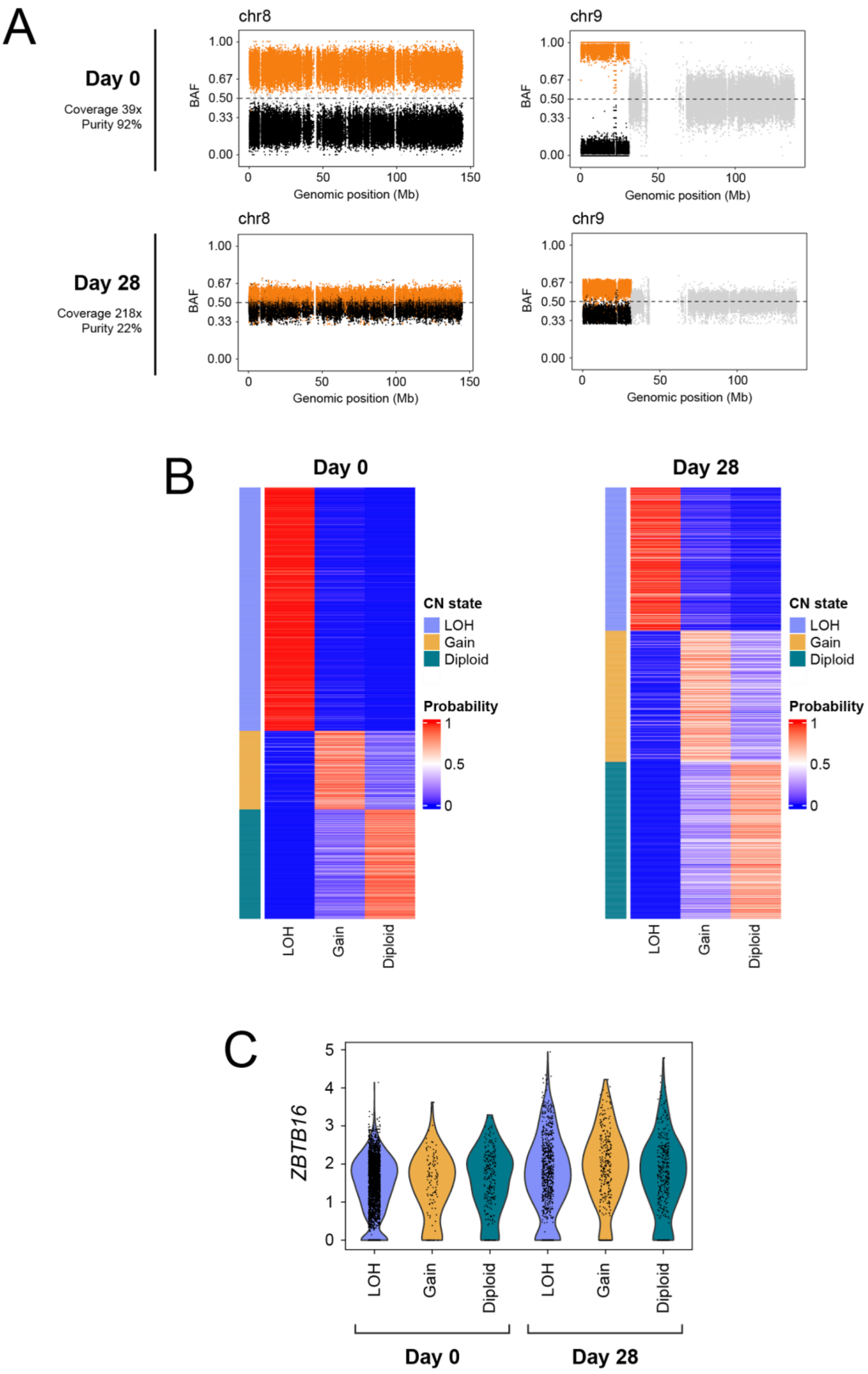

Supplementary Fig. 8: Copy number clones in patient P030.

(**A**) Phased B allele frequency (BAF) of heterozygous single nucleotide polymorphisms (SNPs) on chromosome 8 and 9, in whole genome sequencing (WGS) of the day 0 and day 28 samples of patient P030. Each dot denotes a SNP: major allele (orange), minor allele (black) and SNPs lying outside of the copy number altered segment (grey). Chromosome 9 shows a BAF profile that is consistent with a clonal loss-of-heterozygosity (LOH) at 9p. However, the BAF profile of chromosome 8 is not consistent with either a clonal LOH or a clonal gain, suggesting a subclonal copy number alteration.

(**B**) Heatmap showing the posterior probability of each blast (rows) from P030 bearing an LOH, gain or diploid copy number state on chromosome 8. Three clones of blasts (LOH, gain or diploid at chromosome 8) could be seen at both the day 0 and day 28 samples of P030.

(**C**) Violin plot showing *ZBTB16* in blasts from P030, grouped by their copy number state as shown in **Supplementary Fig. 8B**.

BAF, B allele frequency; LOH, loss-of-heterozygosity.
