# Supplementary material for: A non-canonical lymphoblast in refractory childhood T-cell leukaemia
Source: Nat Commun. 2025 Nov 12;16:9397. doi: 10.1038/s41467-025-65049-8 (PMC12612194; doi:10.1038/s41467-025-65049-8)
Supplement: Supplementary file 3 — Reporting Summary [file 41467_2025_65049_MOESM3_ESM.pdf]

nature portfolio

Corresponding author(s): Sam Behjati, David O'Connor

Last updated by author(s): Jul 18, 2025

Reporting Summary

Nature Portfolio wishes to improve the reproducibility of the work that we publish. This form provides structure for consistency and transparency in reporting. For further information on Nature Portfolio policies, see our Editorial Policies and the Editorial Policy Checklist.

Statistics

For all statistical analyses, confirm that the following items are present in the figure legend, table legend, main text, or Methods section.

n/a	Confirmed	
☐	☒	The exact sample size (*n*) for each experimental group/condition, given as a discrete number and unit of measurement
☐	☒	A statement on whether measurements were taken from distinct samples or whether the same sample was measured repeatedly
☐	☒	The statistical test(s) used AND whether they are one- or two-sided<br>*Only common tests should be described solely by name; describe more complex techniques in the Methods section.*
☐	☒	A description of all covariates tested
☐	☒	A description of any assumptions or corrections, such as tests of normality and adjustment for multiple comparisons
☐	☒	A full description of the statistical parameters including central tendency (e.g. means) or other basic estimates (e.g. regression coefficient) AND variation (e.g. standard deviation) or associated estimates of uncertainty (e.g. confidence intervals)
☐	☒	For null hypothesis testing, the test statistic (e.g. *F*, *t*, *r*) with confidence intervals, effect sizes, degrees of freedom and *P* value noted<br>*Give P values as exact values whenever suitable.*
☒	☐	For Bayesian analysis, information on the choice of priors and Markov chain Monte Carlo settings
☒	☐	For hierarchical and complex designs, identification of the appropriate level for tests and full reporting of outcomes
☒	☐	Estimates of effect sizes (e.g. Cohen's *d*, Pearson's *r*), indicating how they were calculated

Our web collection on statistics for biologists contains articles on many of the points above.

Software and code

Policy information about availability of computer code

Data collection	Flow cytometry was run using BD FACSDiva version 9 (BD Biosciences)
Data analysis	Flow cytometry analysis was performed using FlowJo version 10 (BD Biosciences).<br><br>Raw reads from single-cell RNA sequencing were processed using Cell Ranger (v7.0.0), where reads were aligned to the GRCh38 human reference genome. Ambient mRNA contamination was removed with SoupX (v1.6.2) and doublets were removed with Scrublet (v0.2.3). Downstream analysis of scRNA-seq data was performed by Scanpy (v1.10.0). Differential gene expression analysis was done using edgeR (v3.42.4).<br><br>Raw reads from bulk RNA sequencing were aligned to the GRCh38 reference human genome using STAR (v2.7.10) and quantified with featureCounts (v2.0.2). Downstream analysis of bulk RNA-seq data was performed using edgeR (v3.42.4). Gene module scoring of bulk RNA-seq samples was performed using singscore (v1.20). Survival analyses were performed with the survival R package (v3.5). Normal-to-leukaemia single-cell transcriptome comparison by logistic regression was performed using custom code found here (https://github.com/constantAmateur/scKidneyTumors).<br><br>Raw reads from single-cell TCR sequencing were aligned to the Cell Ranger VDJ reference (v7.0.0), using Cell Ranger (v7.0.0), and subsequently analysed by Dandelion (v0.3.6). TCR analysis from scRNA-seq reads was done using TRUST4 (v0.3.6).<br><br>DNA sequencing reads were aligned to the GRCh38 Ensembl 103 reference genome using the Burrows-Wheeler Alignment tool (v0.7.17). All classes of somatic variants were called using the extensively validated pipeline of the Wellcome Sanger Institute, built on the following

algorithms: CaVEMan (v1.18.2) for base substitutions, Pindel (v3.10.0) for insertions/deletions, ASCAT (v4.5.0) and Battenberg (v3.5.3) for copy number alterations, and BRASS (v6.3.4) and GRIDSS2 (v2.13.1) for structural variants. Copy number alterations in samples which did not have a matched germline sample were called using PURPLE (v3.8.4). The detection of copy number alterations in single-cell transcriptomes was performed using alleleIntegrator (v0.9.1).

Code used in this study are available here (https://github.com/BehjatiLab/T-ALL_refractory).

For manuscripts utilizing custom algorithms or software that are central to the research but not yet described in published literature, software must be made available to editors and reviewers. We strongly encourage code deposition in a community repository (e.g. GitHub). See the Nature Portfolio guidelines for submitting code & software for further information.

Data

Policy information about availability of data

All manuscripts must include a data availability statement. This statement should provide the following information, where applicable:

- Accession codes, unique identifiers, or web links for publicly available datasets
- A description of any restrictions on data availability
- For clinical datasets or third party data, please ensure that the statement adheres to our policy

Sequencing data for the original cohort (DNA, bulk mRNA, single cell mRNA sequencing) are available through the European Genome-Phenome Archive (EGA) under accession codes EGAD00001009058, EGAS50000000718 and EGAD00001015381 (P058 day 28 sample WGS). Processed and raw counts for all single cell mRNA sequencing data (original and validation cohorts) are freely available on the CellxGene repository (https://cellxgene.cziscience.com/collections/962df42d-9675-4d05-bc75-597ec7bf4afb). Raw sequences and processed counts of bulk transcriptomes from the Princess Maxima Center cohort are available from Frank van Leeuwen (f.n.vanleeuwen@prinsesmaximacentrum.nl). Bulk mRNA sequencing counts of the COG AALL0434 cohort (Pölönen et al, 2024) are available for download from the Synapse portal (https://www.synapse.org/Synapse:syn54032669/wiki/627818). Seurat counts for single cell mRNA sequencing data from Xu & Chen et al. (2024) were provided by the authors and raw data are available for download through dbGaP under the accession number phs003432 as part of the Childhood Cancer Data Initiative. Source data are provided with this paper.

Human research participants

Policy information about studies involving human research participants and Sex and Gender in Research.

Reporting on sex and gender	Study results do not apply to any one sex or gender. Sex or gender were not considered in the study design. The sex of patients were noted in Supplementary Data 1.
Population characteristics	Children diagnosed with T-ALL.
Recruitment	Samples were obtained from the VIVO Biobank, Great Ormond Street Haematology Cell Bank and the diagnostic archives of the Great Ormond Street Hospital. The patient cohort represents the typical spectrum of T-ALL subtypes.
Ethics oversight	We accessed tissues from studies approved by UK NHS research ethics committees. The tissue sources were: VIVO Biobank (National Research Ethics Service reference 16SW0219; VIVO project number 23-VIVO-17); Great Ormond Street Haematology Cell Bank (National Research Ethics Service reference 16/LO/0960); samples from the diagnostic archives of Great Ormond Street Hospital (National Research Ethics Service reference 16/EE/0394). Patients or guardians provided informed written consent for participation in this study as stipulated by the study protocols.

Note that full information on the approval of the study protocol must also be provided in the manuscript.

Field-specific reporting

Please select the one below that is the best fit for your research. If you are not sure, read the appropriate sections before making your selection.

[x] Life sciences [ ] Behavioural & social sciences [ ] Ecological, evolutionary & environmental sciences

For a reference copy of the document with all sections, see nature.com/documents/nr-reporting-summary-flat.pdf

Life sciences study design

All studies must disclose on these points even when the disclosure is negative.

Sample size	Sample size calculation was not performed as all samples with sufficient material were sequenced and analysed.
Data exclusions	Bulk RNA sequencing samples from the COG AALL0434 cohort which had less than 60% blasts were excluded from analysis.
Replication	Where possible and relevant, all samples were sequenced once for each modality (scRNA-seq, scTCR-seq, bulk RNA-seq, WGS). For the day 0 sample of L086, two technical replicates of scRNA-seq was performed.

Randomization	Randomisation was not required as no interventions were performed in this study.
Blinding	Blinding was not required as there were no measurements or interpretation that could have been influenced by prior knowledge of results.

Reporting for specific materials, systems and methods

We require information from authors about some types of materials, experimental systems and methods used in many studies. Here, indicate whether each material, system or method listed is relevant to your study. If you are not sure if a list item applies to your research, read the appropriate section before selecting a response.

Materials & experimental systems

n/a	Involved in the study
☐	☒ Antibodies
☒	☐ Eukaryotic cell lines
☒	☐ Palaeontology and archaeology
☒	☐ Animals and other organisms
☒	☐ Clinical data
☒	☐ Dual use research of concern

Methods

n/a	Involved in the study
☒	☐ ChIP-seq
☐	☒ Flow cytometry
☒	☐ MRI-based neuroimaging

Antibodies

Antibodies used

The following antibodies were used (clone; volume; manufacturer; catalogue number) as described in Supplementary Data 16:

CD1a BV421 (HI149; 3uL; BD Biosciences; 563938)
CD2 FITC (RPA-2.10; 2uL; Biolegend; 300206)
CD3 BV785 (OKT3; 3uL; Biolegend; 317330)
CD4 FITC (OKT4; 1uL; Biolegend; 317408)
CD5 PerCP-Cy5.5 (UCHT2; 2uL; Biolegend; 300620)
CD7 APC (CD7-6B7; 2uL; ThermoFisher; MHCD0705)
CD8 BV510 (SK1; 2uL; Biolegend; 344732)
CD11b PECy7 (ICRF44; 2uL; Biolegend; 301322)
CD13 PE (WM15; 2uL; Biolegend; 301704)
CD15 BV510 (W6D3; 3uL; Biologend; 323028)
CD33 BV605 (P67.6; 2uL; Biolegend; 366612)
CD34 PerCP-Cy5.5 (581; 3uL; Biolegend; 343522)
CD45 AF700 (HI30; 2uL; 2uL; Biolegend; 304024)
CD117 BV785 (104D2; 2uL; Biolegend; 313238)
HLA-DR BV421 (L243; 2uL; Biolegend; 307636)
ZBTB16 PE (R17-809; 3uL; BD Biosciences; 564850)

Validation

Antibodies were validated by the manufacturer.

Flow Cytometry

Plots

Confirm that:

☒ The axis labels state the marker and fluorochrome used (e.g. CD4-FITC).

☒ The axis scales are clearly visible. Include numbers along axes only for bottom left plot of group (a 'group' is an analysis of identical markers).

☒ All plots are contour plots with outliers or pseudocolor plots.

☒ A numerical value for number of cells or percentage (with statistics) is provided.

Methodology

Sample preparation

Flow cytometry to ascertain ETP status:
Samples were analysed by flow cytometry to ascertain ETP status as per the reported definition. Once thawed, cells were washed and suspended in flow cytometry buffer (PBS with 2% fetal bovine serum) and stained with antibodies at 4ºC in the dark for 30 minutes.

Intra-cytoplasmic flow cytometry assessment of ZBTB16:
Once thawed, cells were fixed and permeabilised using the BD Cytofix/Cytoperm™ Fixation/Permeabilization Kit as per

	manufacturer's instructions. Briefly, cells were washed and suspended in flow buffer and stained with antibodies to surface antigens, including CD3 BV785 (Biolegend), CD45 AF700 (Biolegend) and CD7 APC (Thermo Fisher Scientific) for 30 minutes at 4°C. Cells were washed and resuspended in Fixation/Permeabilization solution for 20 minutes at 4°C. Cells were then washed and resuspended in BD Perm/Wash™ buffer containing the ZBTB16 (PLZF) PE antibody (BD) for 30 minutes at 4° C. Control cells were incubated with an appropriate Mouse PE isotype control (BD). Following a final wash, cells were resuspended in flow cytometry buffer.
Instrument	Flow cytometry was performed on a BD Fortessa X-20 flow cytometer.
Software	Flow cytometry was run using BD FACSDiva version 9 (BD Biosciences) and analysed using FlowJo version 10 (BD Biosciences).
Cell population abundance	Cell population abundances are shown in Supplementary Data 3.
Gating strategy	ETP status was determined by the standard definition as reported in the main text.

☐ Tick this box to confirm that a figure exemplifying the gating strategy is provided in the Supplementary Information.
