# Supplementary material for: A non-canonical lymphoblast in refractory childhood T-cell leukaemia
Source: Nat Commun. 2025 Nov 12;16:9397. doi: 10.1038/s41467-025-65049-8 (PMC12612194; doi:10.1038/s41467-025-65049-8)
Supplement: Supplementary file 4 — Description of Additional Supplementary Files [file 41467_2025_65049_MOESM4_ESM.pdf]

Description of Additional Supplementary Files

Supplementary Data 1. Patient cohort (original and validation scRNA-seq cohort).

Supplementary Data 2. Driver mutation matrix.

Supplementary Data 3. Diagnostic flow cytometry profiles.

Supplementary Data 4. Scanpy comparison of P058 and P030 day 28 blasts against all other blasts to obtain the *ZBTB16* marker gene.

Supplementary Data 5. Scanpy comparison of all day 28 refractory blasts against all day 0 responsive blasts.

Supplementary Data 6. Differential gene expression to derive gene module.

Supplementary Data 7. TCR gene usage of T-ALL blasts.

Supplementary Data 8. Functional annotation of genes in gene module.

Supplementary Data 9. Clinical details of Princess Maxima Center cohort with signals of *ZBTB16*+ blasts.

Supplementary Data 10. Clinical details of COG AALL0434 cohort with signals of *ZBTB16*+ blasts.

Supplementary Data 11. Cox proportional hazards models testing *ZBTB16* and immunophenotype-defined ETP status on the COG AALL0434 cohort.

Supplementary Data 12. Cox proportional hazards models testing *ZBTB16* and transcriptome-defined ETP-like status on the COG AALL0434 cohort.

Supplementary Data 13. Cox proportional hazards models testing *ZBTB16* and BMP gene modules on the COG AALL0434 cohort.

Supplementary Data 14. Catalogue of base substitutions in patient P058.

Supplementary Data 15. Functional annotation of cell surface target genes.

Supplementary Data 16. Flow cytometry antibodies.
